# Supplementary material for: Continent-wide genomic signatures of adaptation to urbanisation in a songbird across Europe
Source: Nat Commun. 2021 May 20;12:2983. doi: 10.1038/s41467-021-23027-w (PMC8137928; doi:10.1038/s41467-021-23027-w)
Supplement: Supplementary file 1 — Supplementary Information [file 41467_2021_23027_MOESM1_ESM.pdf]

Supplementary Information to

Continent-wide genomic signatures of adaptation to urbanisation in a songbird across Europe

Pablo Salmón[†], Arne Jacobs[†], Dag Ahrén, Clotilde Biard, Niels J. Dingemanse, Davide Dominoni, Barbara Helm, Max Lundberg, Juan Carlos Senar, Philipp Sprau, Marcel Visser and Caroline Isaksson.

[†] Both authors contributed equally

Supplementary Figures

Supplementary Fig. 1. Proportion of land cover at each sampling site.
Supplementary Fig. 2. Genetic ancestry in urban and rural European great tits.
Supplementary Fig. 3. Principal components analysis and UMAP.
Supplementary Fig. 4. TreeMix migration events.
Supplementary Fig. 5. Extended LFMM results.
Supplementary Fig. 6. BayPass genotype-urbanisation association.
Supplementary Fig. 7. Allele frequency shifts in urbanisation-associated SNPs.
Supplementary Fig. 8. Allele frequency trajectories between urban and rural populations at "Core urbanisation SNPs".
Supplementary Fig. 9. Distribution of minor allele frequency differences.
Supplementary Fig. 10. Intersection of ongoing and recent selective sweep windows across urban populations.
Supplementary Fig. 11. ZF_{ST} Manhattan plots.
Supplementary Fig. 12. Intersection of ZF_{ST} outlier windows
Supplementary Fig. 13. Selective sweep signatures (*Rsb*).
Supplementary Fig. 14. Intersection of *Rsb* outlier windows.
Supplementary Fig. 15. Intersection of genes associated with *XP-nSL* selective sweep signatures.
Supplementary Fig. 16. Intersection of genes associated with *Rsb* selective sweep signatures.
Supplementary Fig. 17. Network of urbanisation associated (LFMM) gene ontology (GO) terms (biological processes).

Supplementary Tables

Supplementary Table 1. Information on the studied populations.
Supplementary Table 2. Pairwise genetic differentiation among populations.
Supplementary Table 3. Genes associated with "Core urbanisation SNPs".
Supplementary Table 4. Genes putatively under selection in at least five cities based on *XP-nSL* or *Rsb*.
Supplementary Table 5. Overrepresented GO terms for genes associated with urbanisation in the LFMM and BayPass analysis.

Supplementary Figures

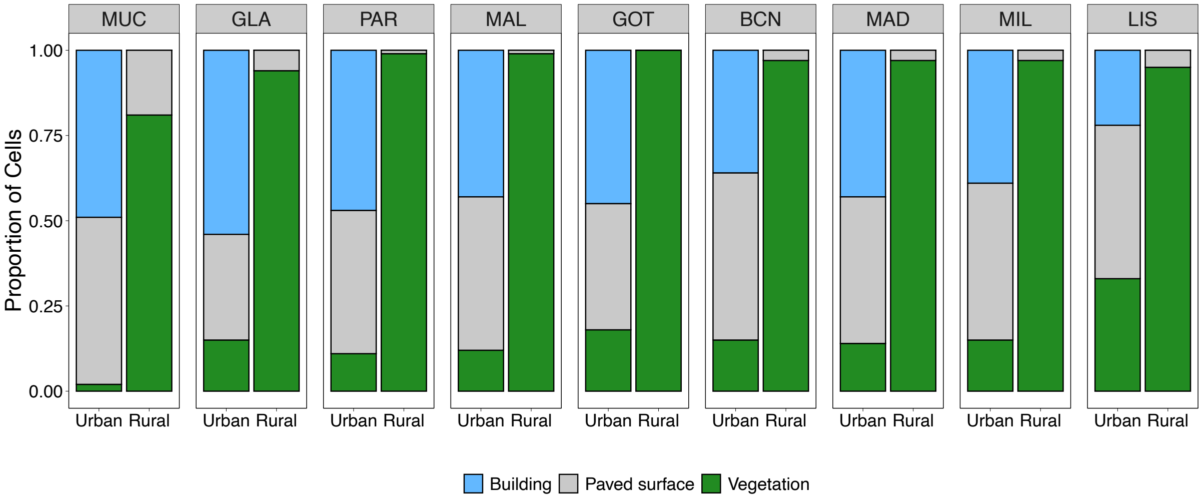

Supplementary Fig. 1. Proportion of land cover at each sampling site. Proportion of cells (100 x100 m) considering three land-cover characteristics: proportion of buildings, paved surfaces and vegetation (including cultivated fields), in each of the sampling sites (Urban/Rural) per urban-rural pair (city). Data acquired using the UrbanizationScore image-analysis software and based on aerial images from Google Maps (Google Maps 2017). Sampling sites sorted according to Urbanisation scores (principal component, PC_{urb}) from higher to lower score. BCN: Barcelona; GLA: Glasgow; GOT: Gothenburg; LIS: Lisbon; MAD: Madrid; MAL: Malmö; MIL: Milan; MUC: Munich; PAR: Paris. Source data are provided as a Source Data file.

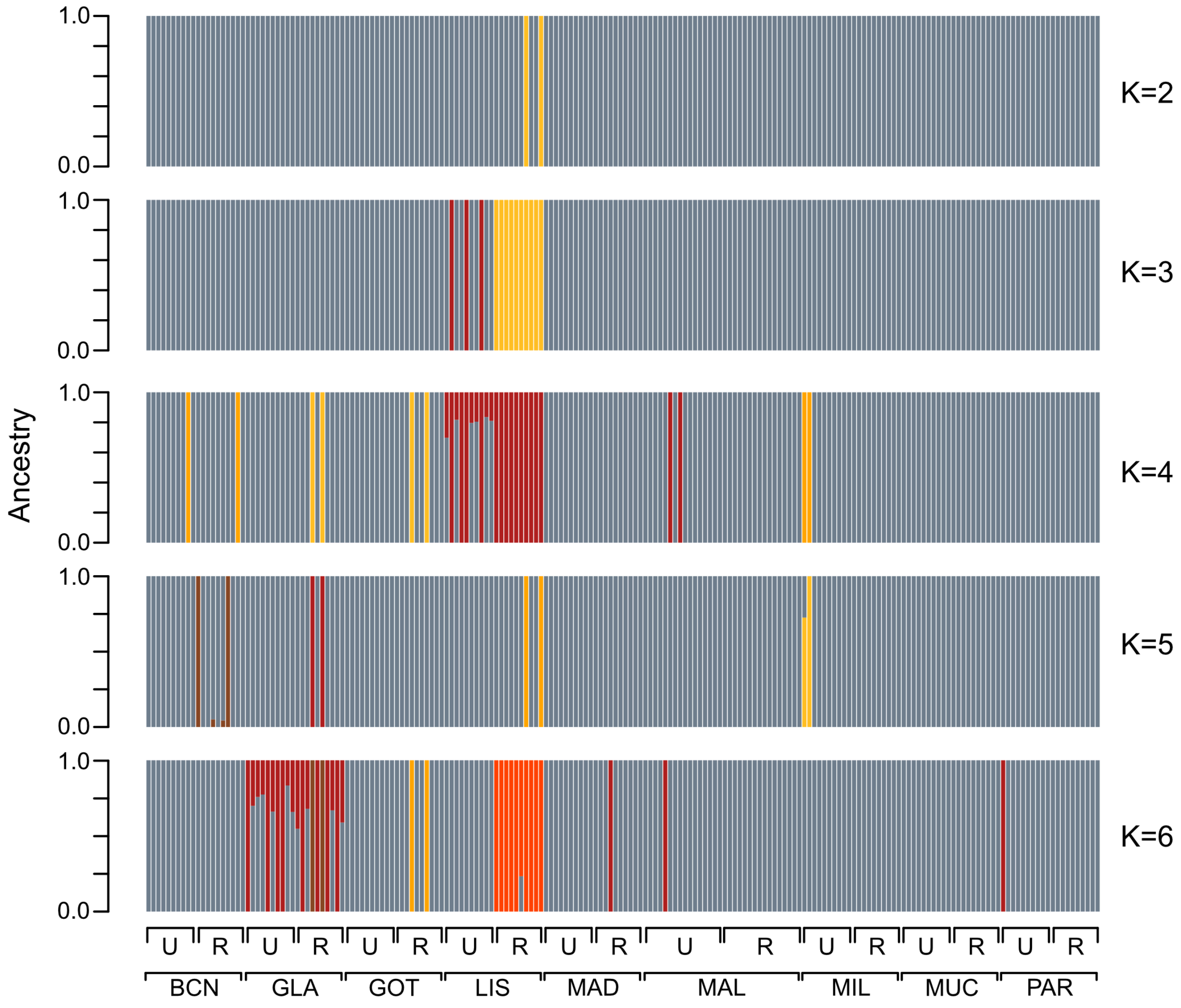

Supplementary Fig. 2. Genetic ancestry in urban and rural European great tits. Genetic ancestry was inferred with fastStructure for K=2 to K=9. The lowest cross-validation error was obtained with K=4 (CV = 0.589). BCN: Barcelona; GLA: Glasgow; GOT: Gothenburg; LIS: Lisbon; MAD: Madrid; MAL: Malmö; MIL: Milan; MUC: Munich; PAR: Paris.

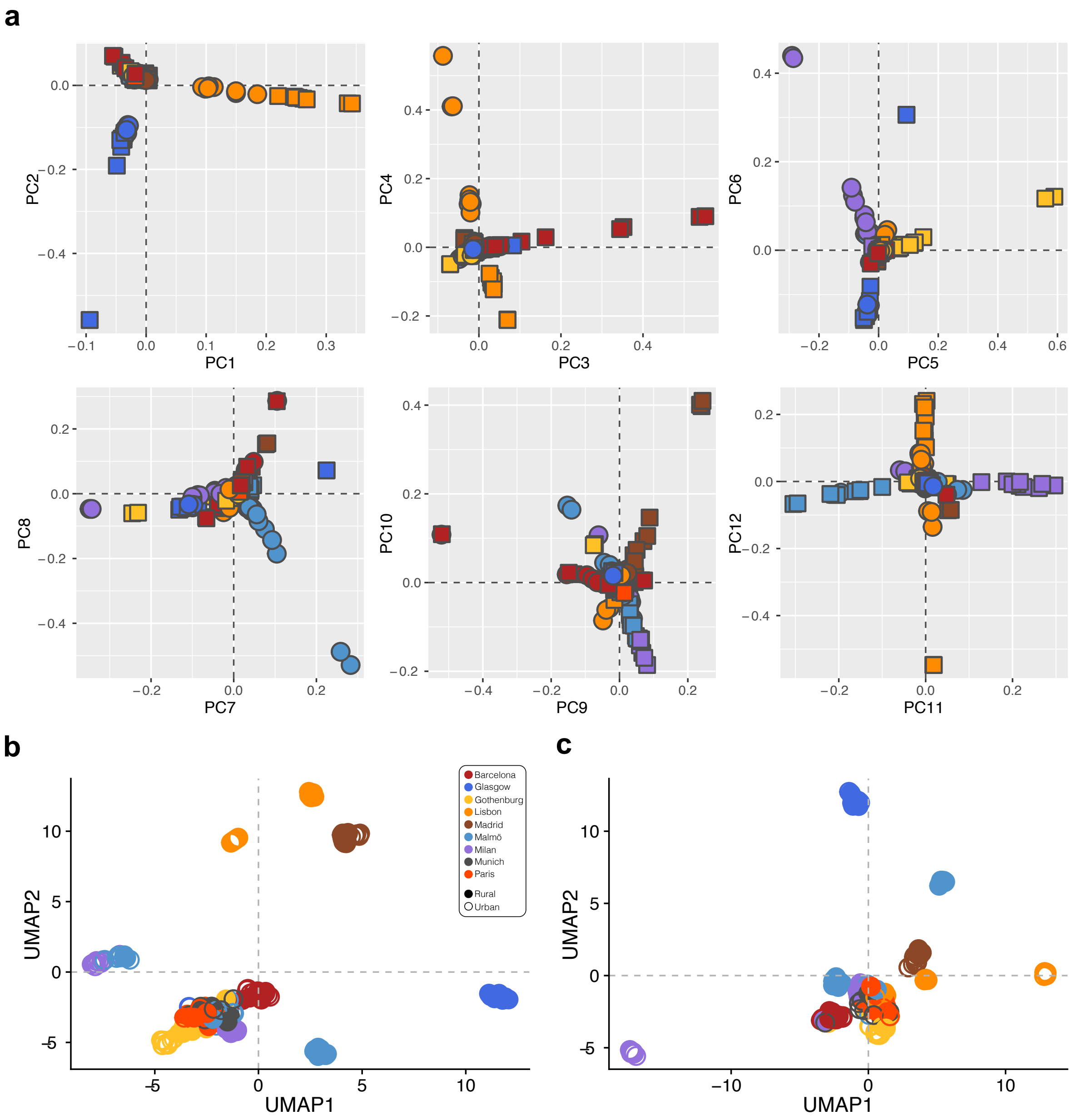

Supplementary Fig. 3. Principal components analysis and UMAP. a, PCA plots for PC1 to PC12 based on an LD-pruned dataset excluding the Z-chromosome and small linkage groups. **b,** UMAP reduction of the first 10 PCs shown in panel "***a***". **c,** UMAP reduction of the first twenty PCs of a PCA including the Z-chromosome. The population clustering is very similar compared to the UMAP plot without the Z-chromosome.

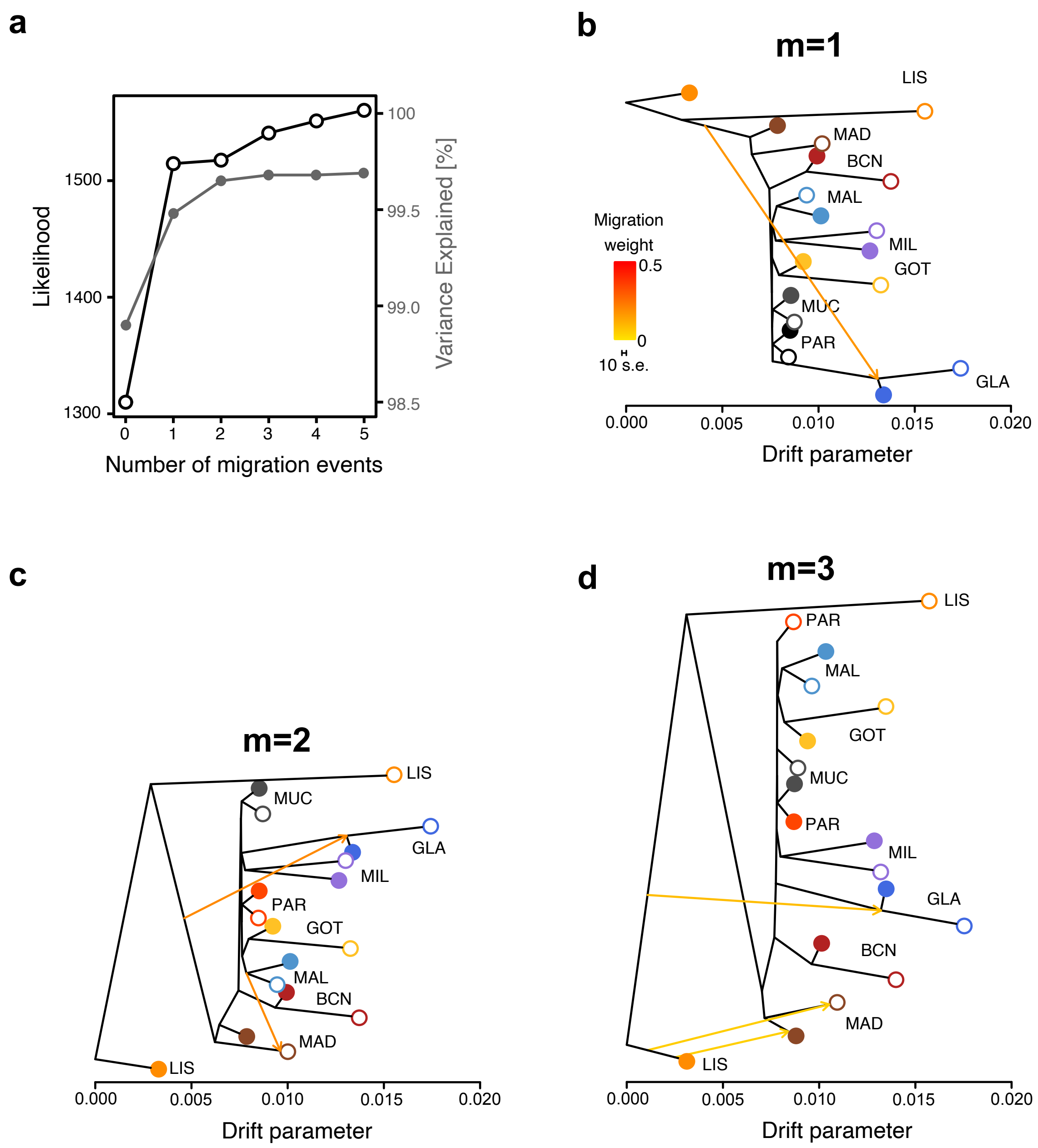

Supplementary Fig. 4. TreeMix migration events. a, Likelihoods (open circles, black) and Variance Explained (closed circles, grey) for TreeMix population trees with different numbers of fitted migration events, from m=0 to m=5. **b, c, d,** TreeMix population trees with **b:** m=1, **c:** m=2 and **d:** m=3 fitted migration events. Migration events and their direction are indicated by arrows, with the colour of the arrow indicating the migration weight (or strength of gene flow). We determined m=2 as the optimal number of migration edges, as *i)* the Variance Explained starts to plateau, *ii)* the likelihood shows an initial plateau and *iii)* one of the fitted migration edges at m=3 is non-significant. Open circles represent urban populations and closed circles rural populations. BCN: Barcelona; GLA: Glasgow; GOT: Gothenburg; LIS: Lisbon; MAD: Madrid; MAL: Malmö; MIL: Milan; MUC: Munich; PAR: Paris.

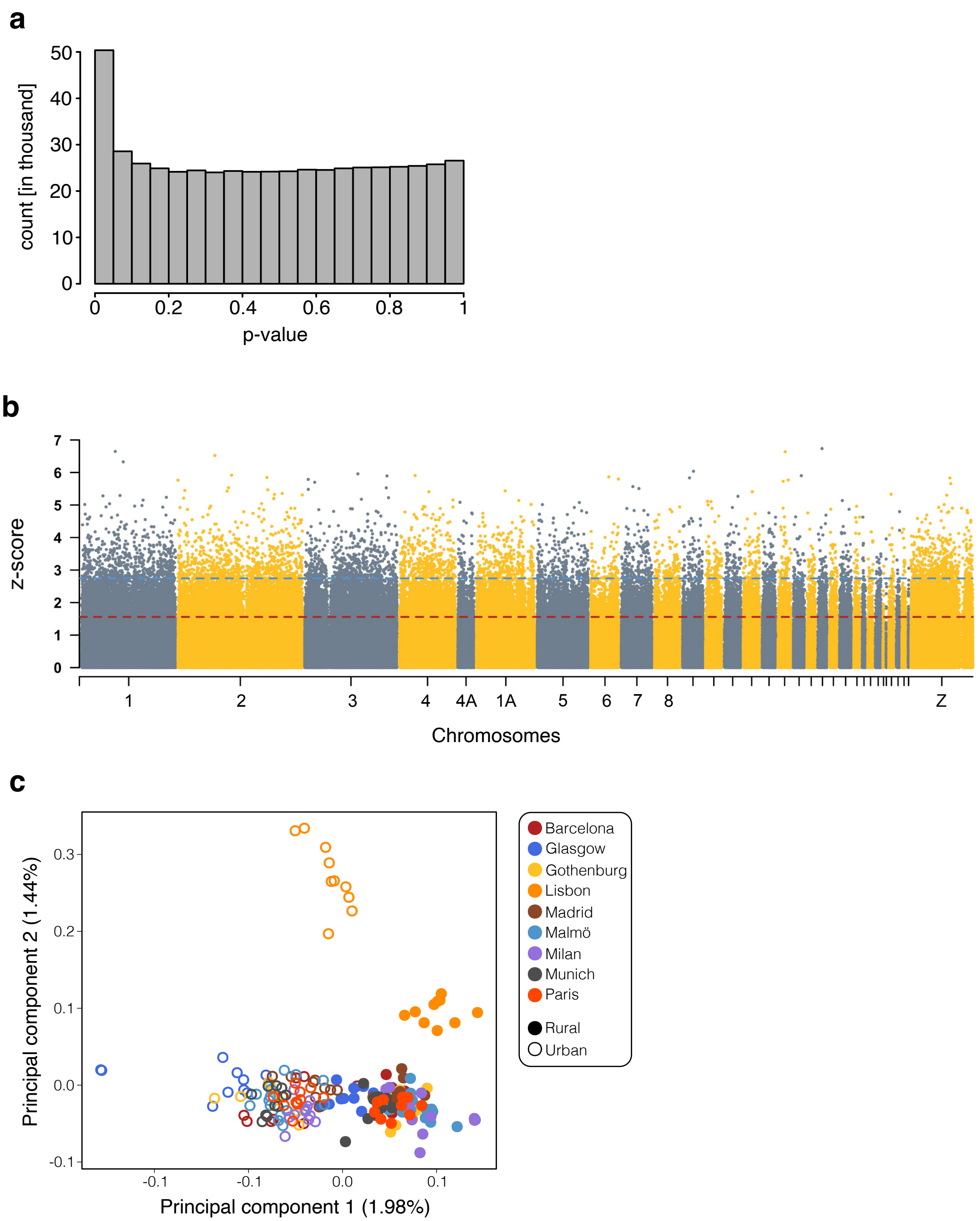

Supplementary Fig. 5. Extended LFMM results. a, Distribution of p-values from LFMM. **b,** Manhattan plot of z-scores from LFMM. The red and blue dotted lines depict the 95th and 99th percentile cut-offs derived from the permutation analysis. **c,** PCA based on all LFMM urbanisation-associated SNPs based on an FDR below 1%. Latent Factor Mixed models (**a**).

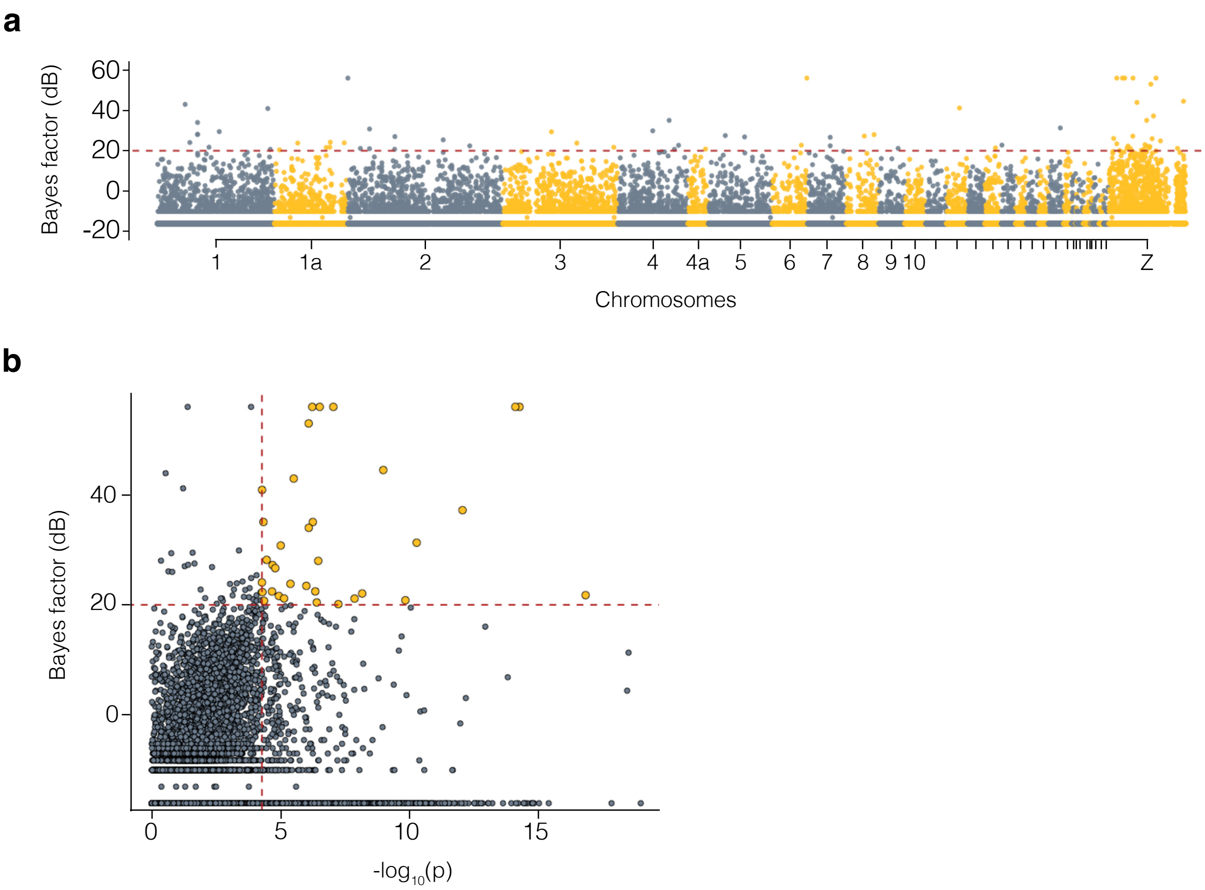

Supplementary Fig. 6. BayPass genotype-urbanisation association. a, Manhattan plot depicting the association between genotype and urbanisation score for each SNP across all populations. The red-dashed line shows the threshold of 20dB that indicates strong evidence for association (see Methods for details). **b,** Intersection between p-values from LFMM (x-axis) and Bayes factors from BayPass for genotype-urbanisation associations. Shared significantly associated SNPs are highlighted in yellow. The red dashed lines indicate the respective significance thresholds.

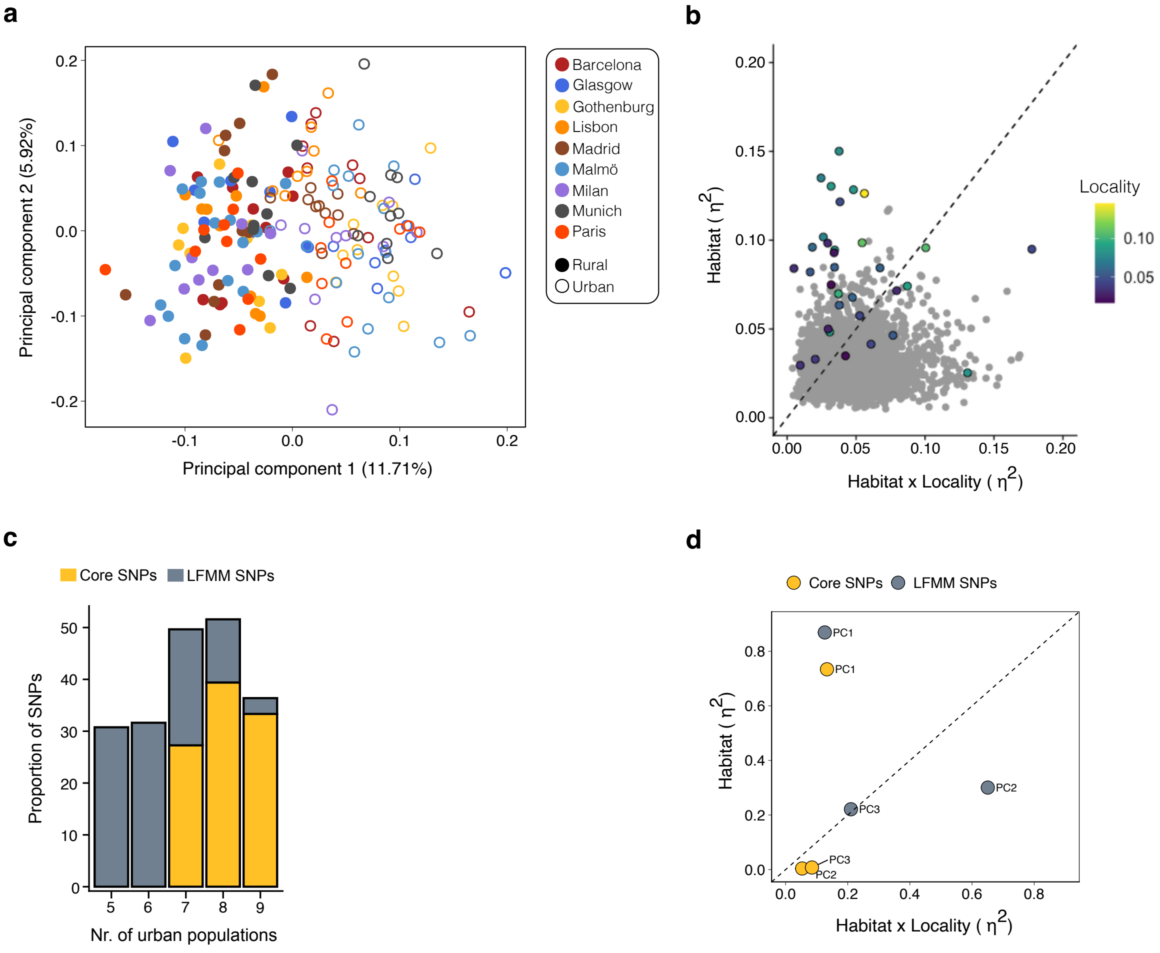

Supplementary Fig. 7. Allele frequency shifts in urbanisation-associated SNPs. a, First two principal component axis are shown (Percentage of total variance explained) for a PCA based on shared "Core urbanisation SNPs" between LFMM and BayPass (n=34). **b,** For each urbanisation-associated SNP (from LFMM) we estimated the effect sizes (partial η^2) for the effects of "Habitat" (urban vs rural), "Locality" (cities) and "Habitat x Locality" interaction on allele frequencies using linear models. The effect size for "Habitat x Locality" interaction and "Habitat" are shown for each SNP on the x-axis and y-axis, respectively, with the "Habitat" effect indicating consistent allele frequencies differences across localities while the interaction term indicates inconsistent changes across localities. SNPs that lie above the dashed line show highly consistent shifts in allele frequencies across localities, whereas SNPs below the line show inconsistent shifts in allele frequency across localities. "Core-urbanisation SNPs" are highlighted and coloured depending on the effect of locality on allele frequency shifts (see Legend in figure), with the locality effect highlighting absolute differences in allele frequency differences across localities. **c,** Bar plot showing the proportion of urbanisation-associated SNPs (LFMM and "Core-urbanisation SNPs") that show consistent differences in allele frequency between urban and rural populations across all localities. For example, SNPs for which the same allele is the minor allele in all urban populations have an x-value of 9, whereas those that only have the same minor allele in 5 of the populations (the lowest possible by chance) have a x-value of 5. Note the right-skew of all associated SNPs, but particularly of the "Core-urbanisation SNPs", indicating highly consistent allele frequency differences across Europe. **d,** In addition to estimate the effect sizes for the "Habitat" and "Habitat x Locality" interaction for each individual SNP (see panel "**b**"), we also estimated their effect sizes using the same linear models for the first three principal component

axes from PCAs for all significant LFMM SNPs and only "Core-urbanisation SNPs". Note that PC1 explains the majority of the genetic variance and in both cases shows a stronger overall effect of "Habitat", supporting our inference of highly consistent allele frequency shifts between urban and rural populations based on individual SNPs.

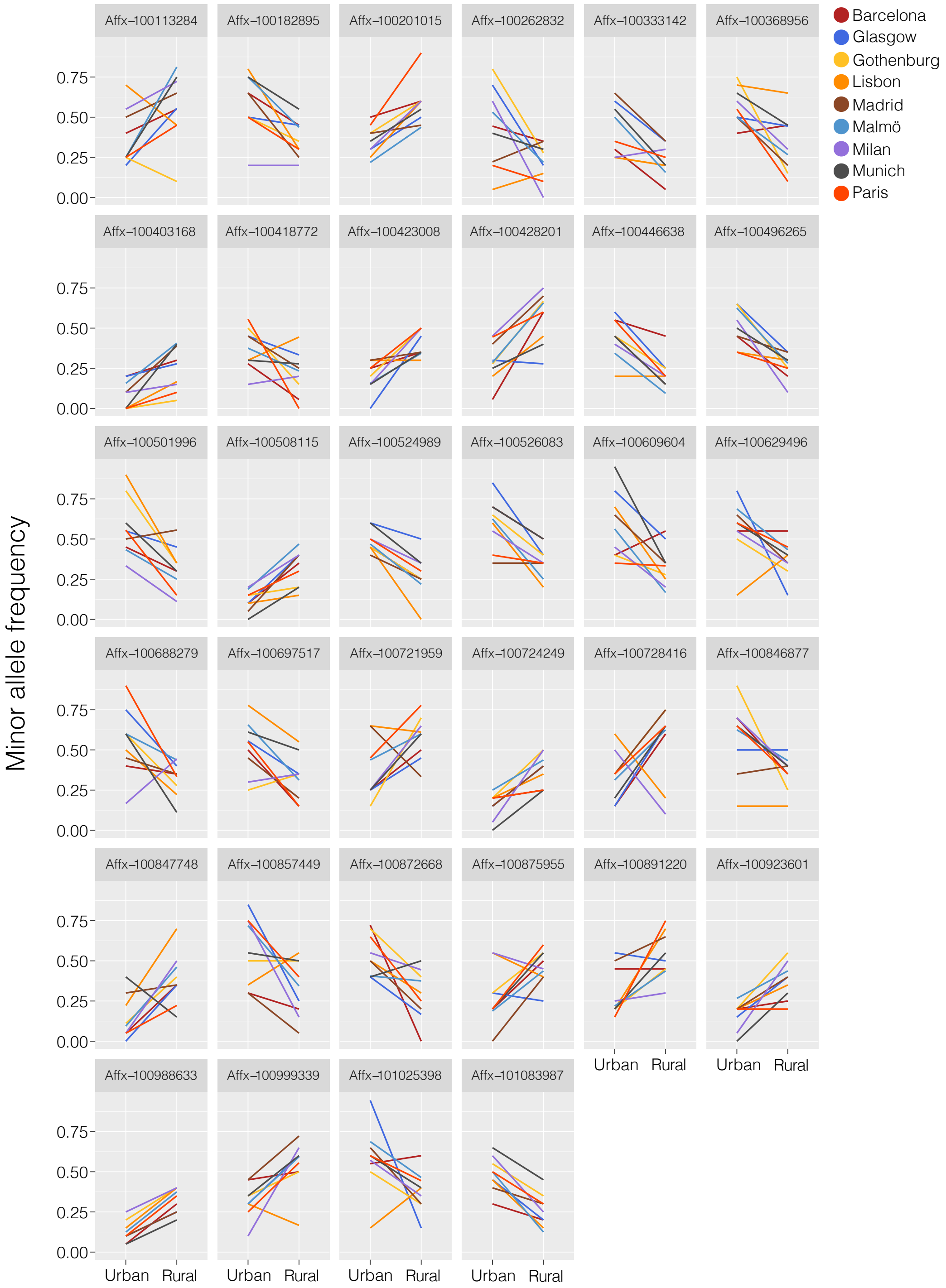

Supplementary Fig. 8. Allele frequency trajectories between urban and rural populations at "Core urbanisation SNPs". Minor allele frequency (MAF) trajectories between urban and rural populations for all "Core urbanisation SNPs" (n = 34). The Affx-number in the header shows the name for each SNP on the Affymetrix SNP-chip used for the genotyping of all individuals in this study. Effect sizes of each variable ("Habitat", "Locality" and "Habitat x Locality" interaction) in the model are shown in Figs. 3d and S7.

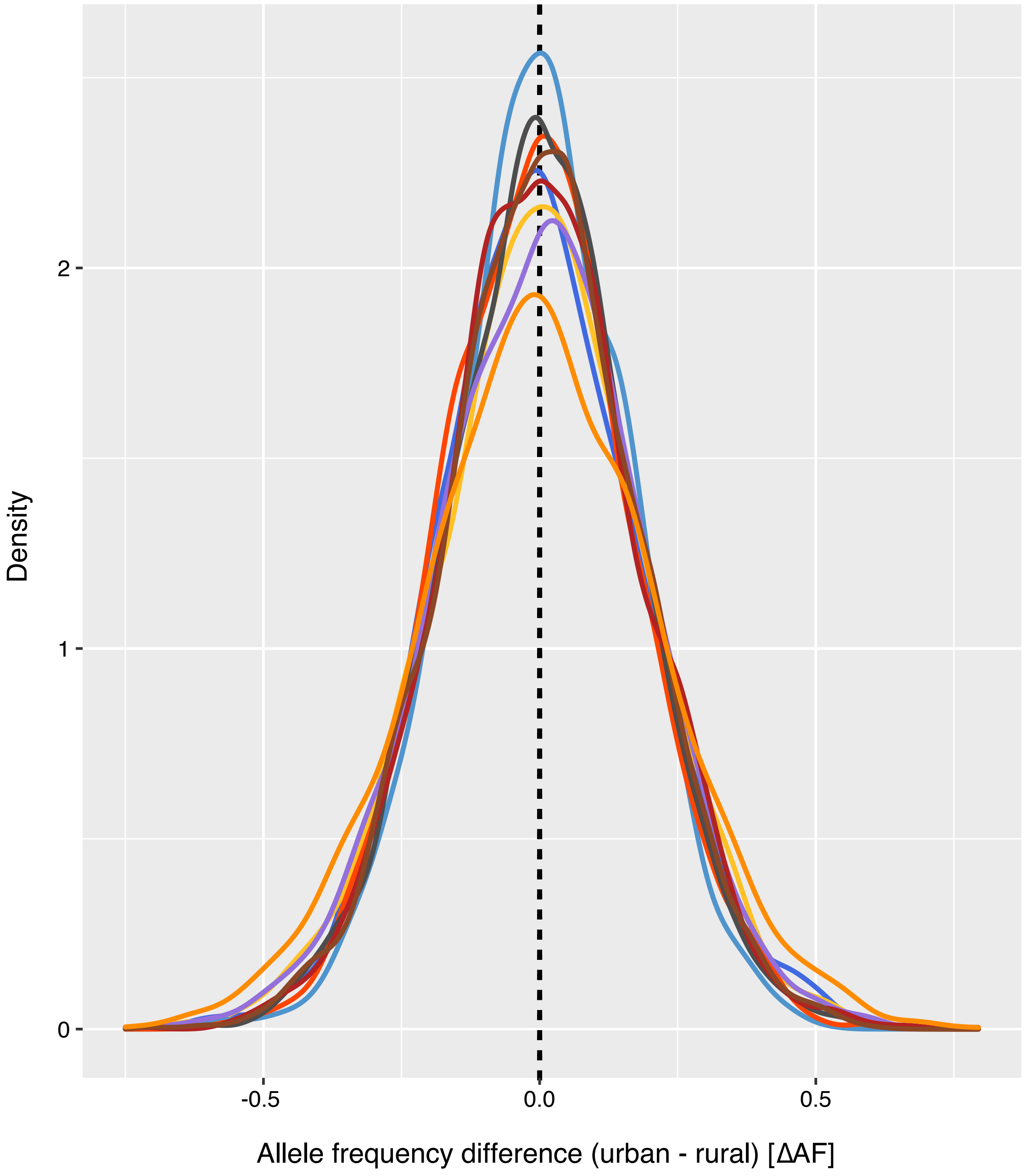

Supplementary Fig. 9. Distribution of minor allele frequency differences [ΔAF] between adjacent urban and rural populations for all urbanisation-associated SNPs from the LFMM analysis. Positive values indicate a higher minor allele frequency in urban populations and *vice versa*.

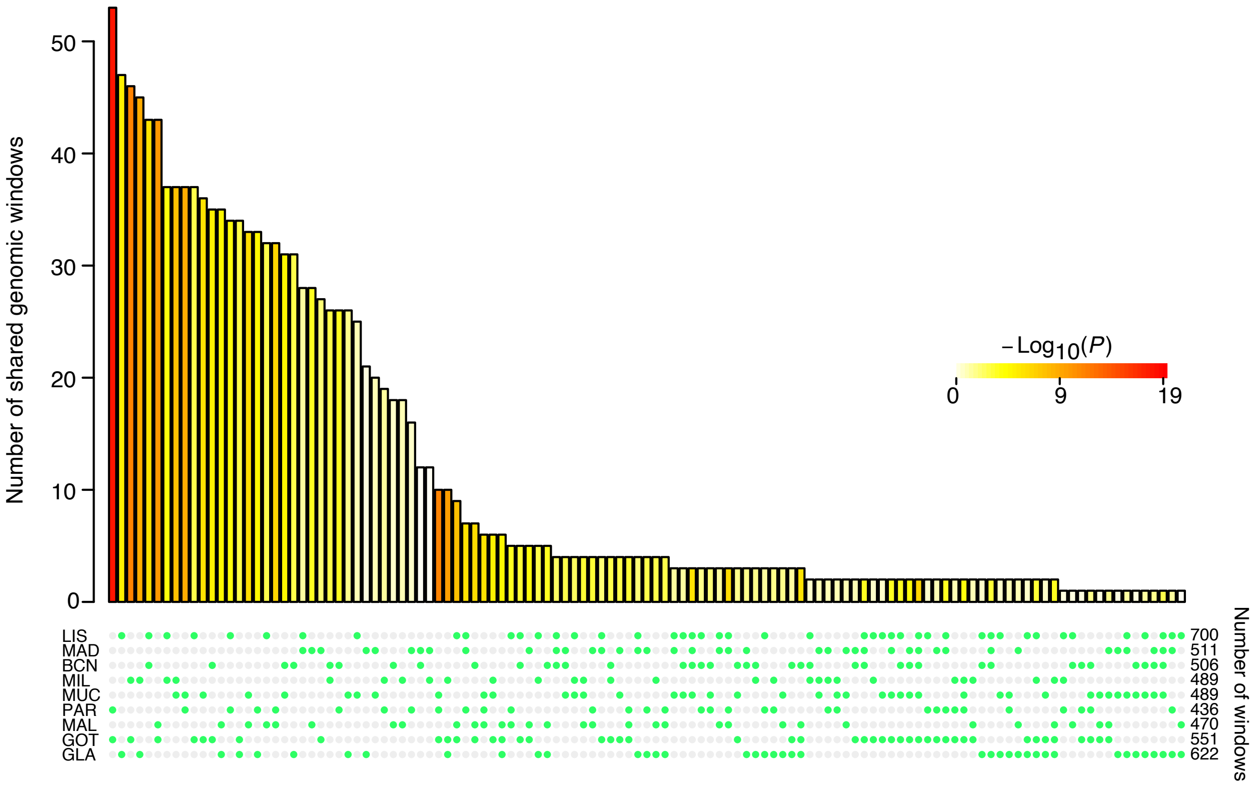

Supplementary Fig. 10. Intersection of ongoing and recent selective sweep windows across urban populations. The intersection of 200kb genomic windows that showed significant signs of selective sweeps based on *XP-nSL*. The bar plot in the top panel shows how many genomic windows are shared for each multi-way comparison, with the populations that are compared highlighted by green dots in the lower panel. The colour of the bar plot indicates the significance of the comparison, i.e., are more windows shared in the respective multi-way comparison than expected by chance (Legend shows respective p-values). The number of windows in the lower panel corresponds to the total number of outlier windows detected in each population. Bonferroni-adjusted p-values were estimated using one-sided Fisher's Exact Tests in the SuperExactTest R-package (see Methods). The population code is indicated on the left in the lower panel (BCN: Barcelona; GLA: Glasgow; GOT: Gothenburg; LIS: Lisbon; MAD: Madrid; MAL: Malmö; MIL: Milan; MUC: Munich; PAR: Paris). Source data are provided as a Source Data file.

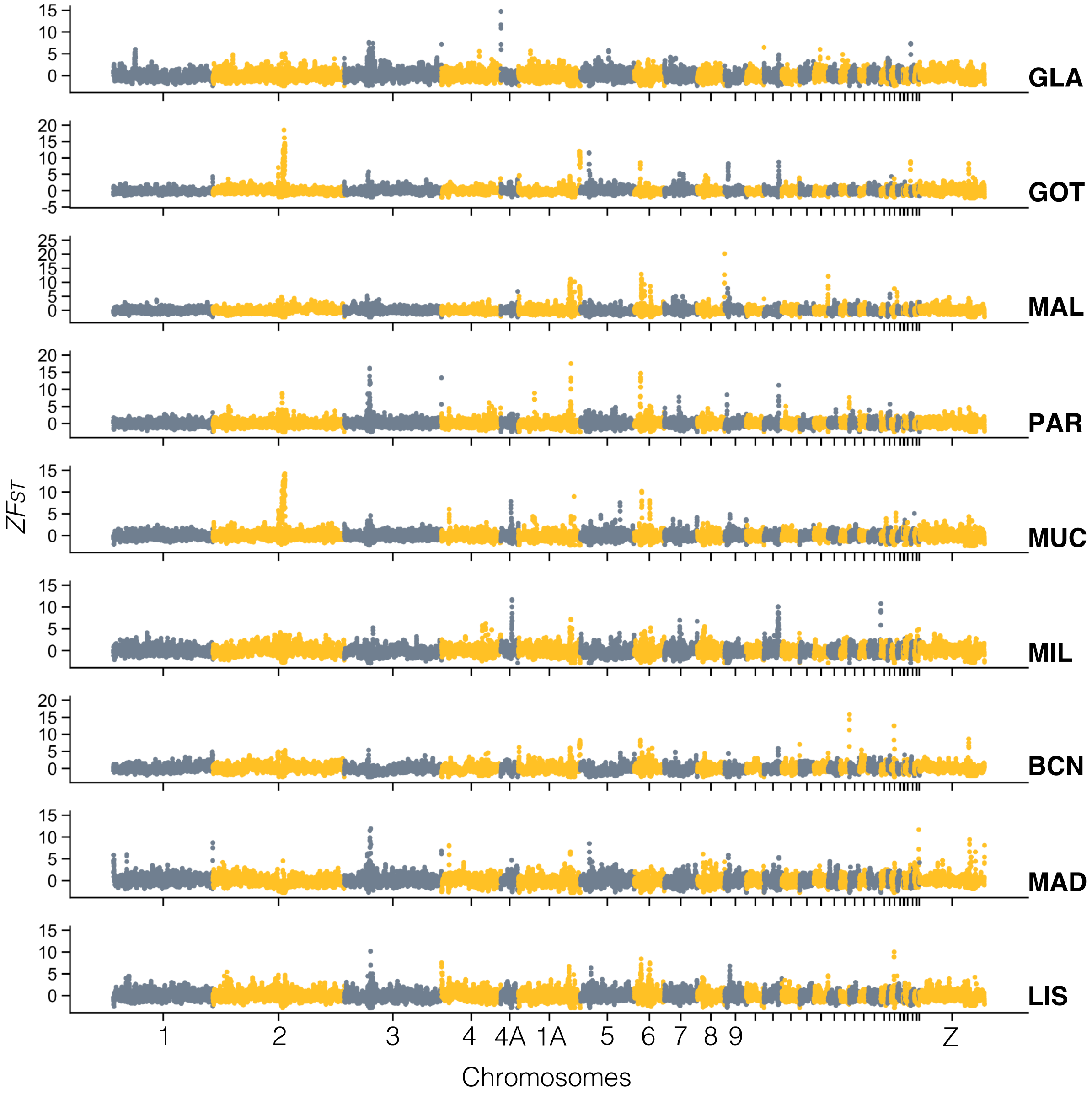

Supplementary Fig. 11. *ZF_{ST}* Manhattan plots. Manhattan plots showing Z-transformed F_{ST} (ZF_{ST}) values (200 kb sliding windows with 50 kb steps) between urban and rural individuals across the genome for each population pair (city). Outlier windows are those with a ZF_{ST} values above 4. BCN: Barcelona; GLA: Glasgow; GOT: Gothenburg; LIS: Lisbon; MAD: Madrid; MAL: Malmö; MIL: Milan; MUC: Munich; PAR: Paris.

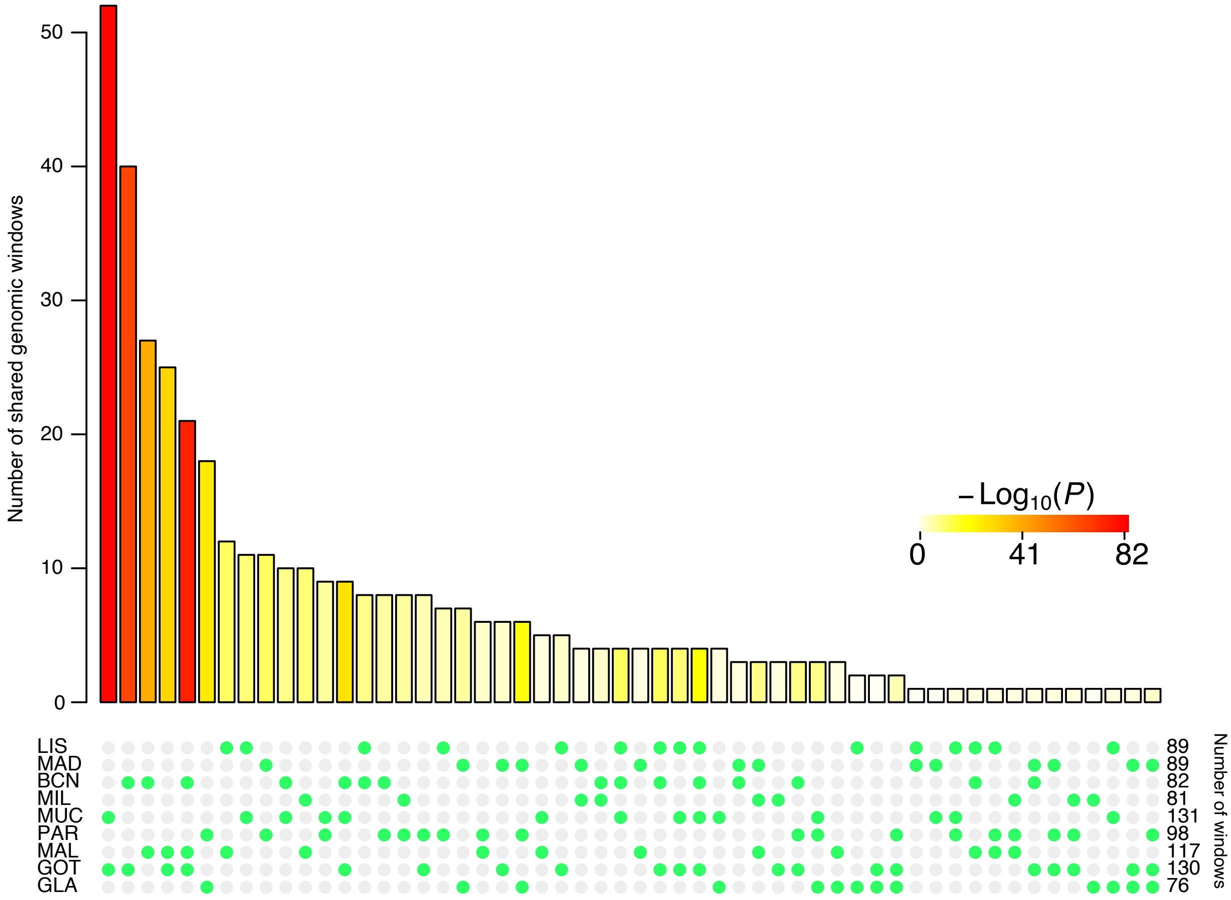

Supplementary Fig. 12. Intersection of ZF_{ST} outlier windows. The intersection of 200kb genomic windows that were significant ZF_{ST} outliers ($ZF_{ST} > 4$). The bar plot in the top panel shows how many genomic windows are shared for each multi-way comparison, with the populations that are compared highlighted by green dots in the lower panel. The colour of the bar plot indicates the significance of the comparison, i.e., are more windows shared in the respective multi-way comparison than expected by chance (Legend shows respective p-values). The number of windows in the lower panel corresponds to the total number of outlier windows detected in each population. Bonferroni-adjusted p-values were estimated using one-sided Fisher's Exact Tests in the SuperExactTest R-package (see Methods). The population code is indicated on the left in the lower panel (BCN: Barcelona; GLA: Glasgow; GOT: Gothenburg; LIS: Lisbon; MAD: Madrid; MAL: Malmö; MIL: Milan; MUC: Munich; PAR: Paris). Source data are provided as a Source Data file.

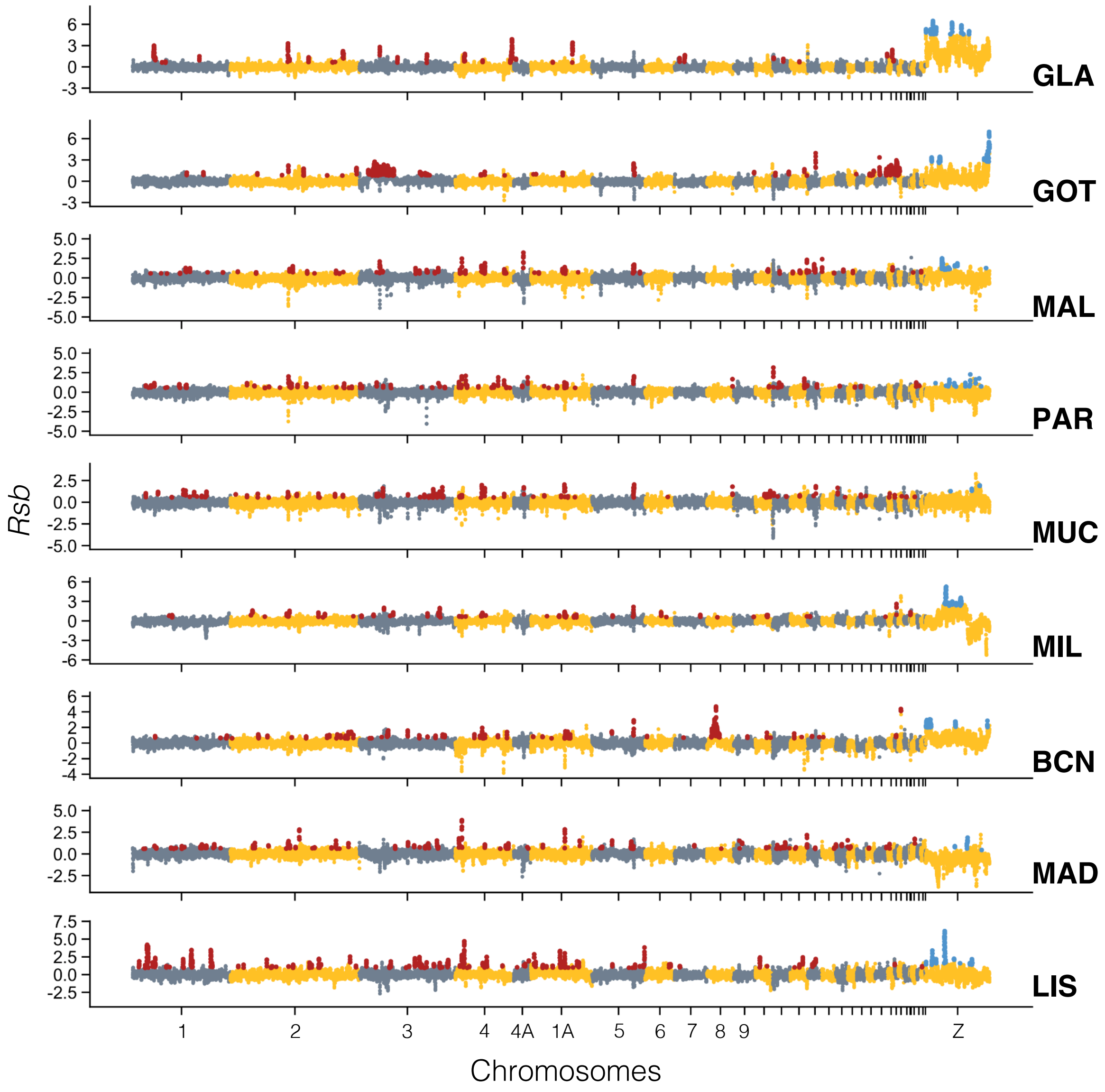

Supplementary Fig. 13. Selective sweep signatures (*Rsb*). Manhattan plots showing haplotype-based *Rsb* selection scores in 200 kb windows with 50 kb steps between urban and rural individuals across the genome for each population pair (urban-rural). Red dots indicate significant autosomal outlier windows with signs of selective sweeps in urban populations, while blue dots are outlier windows detected for the Z-chromosome. BCN: Barcelona; GLA: Glasgow; GOT: Gothenburg; LIS: Lisbon; MAD: Madrid; MAL: Malmö; MIL: Milan; MUC: Munich; PAR: Paris.

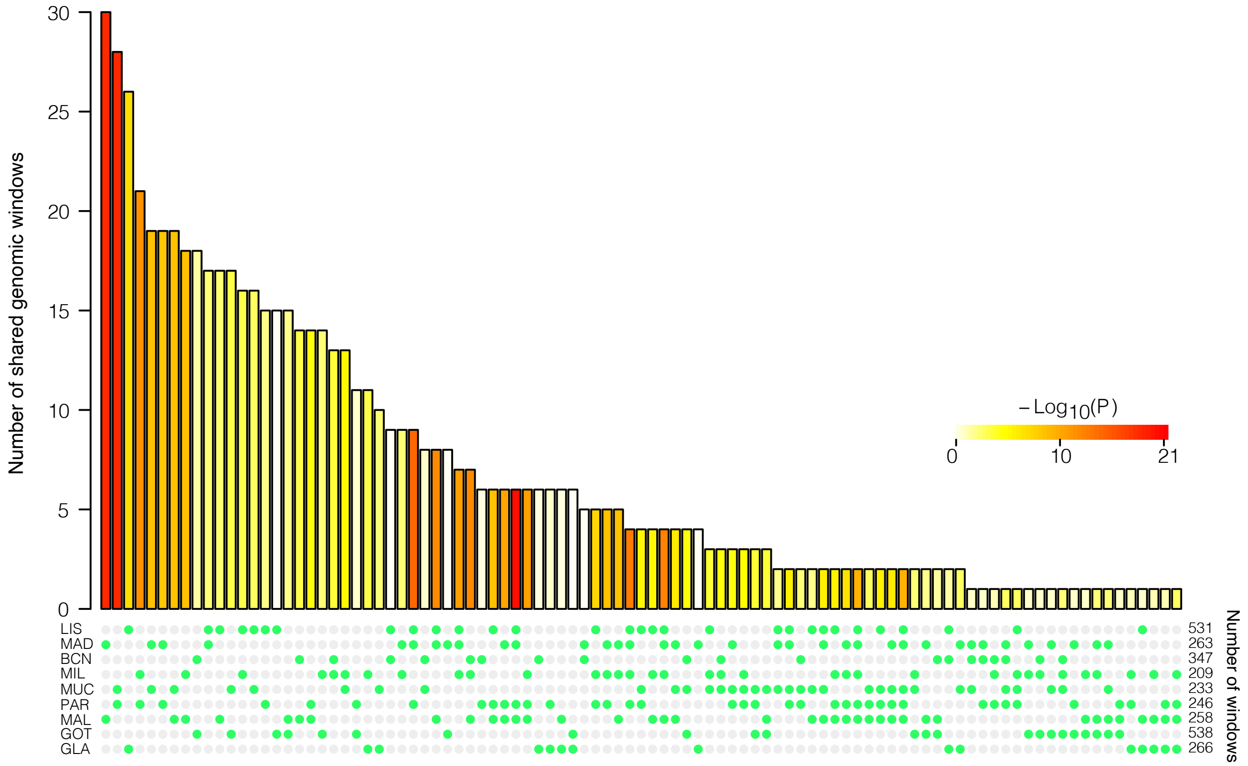

Supplementary Fig. 14. Intersection of *Rsb* outlier windows. The intersection of 200kb genomic windows that showed significant signs of selective sweeps based on *Rsb*. The bar plot in the top panel shows how many genomic windows are shared for each multi-way comparison, with the populations that are compared highlighted by green dots in the lower panel. The colour of the bar plot indicates the significance of the comparison, i.e., are more windows shared in the respective multi-way comparison than expected by chance (Legend shows respective p-values). The number of windows in the lower panel corresponds to the total number of outlier windows detected in each population. Bonferroni-adjusted p-values were estimated using one-sided Fisher's Exact Tests in the SuperExactTest R-package (see Methods). The population code is indicated on the left in the lower panel (BCN: Barcelona; GLA: Glasgow; GOT: Gothenburg; LIS: Lisbon; MAD: Madrid; MAL: Malmö; MIL: Milan; MUC: Munich; PAR: Paris). Source data are provided as a Source Data file.

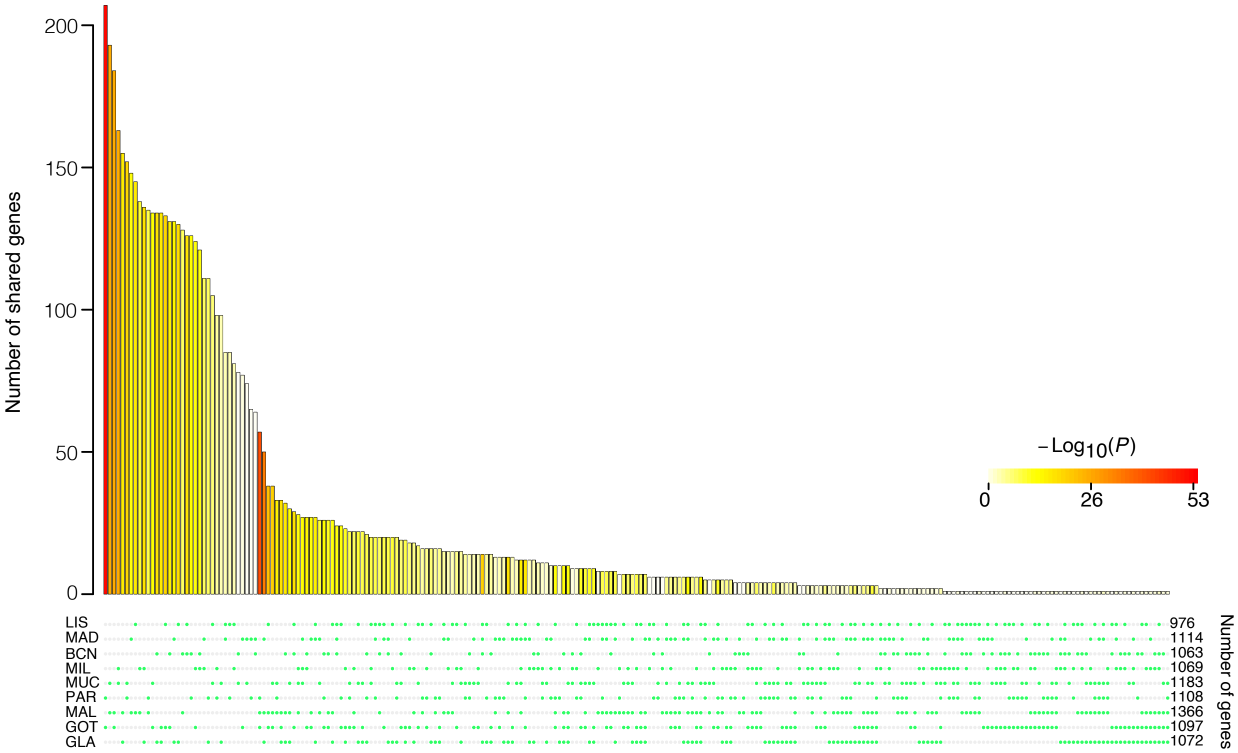

Supplementary Fig. 15. Intersection of genes associated with *XP-nSL* selective sweep signatures. The bar plot in the top panel shows how many genomic windows are shared for each multi-way comparison, with the populations that are compared highlighted by green dots in the lower panel. The colour of the bar plot indicates the significance of the comparison, i.e., are more windows shared in the respective multi-way comparison than expected by chance (Legend shows respective p-values). The number of windows in the lower panel corresponds to the total number of outlier windows detected in each population. Bonferroni-adjusted p-values were estimated using one-sided Fisher's Exact Tests in the SuperExactTest R-package (see Methods). The population code is indicated on the left in the lower panel (BCN: Barcelona; GLA: Glasgow; GOT: Gothenburg; LIS: Lisbon; MAD: Madrid; MAL: Malmö; MIL: Milan; MUC: Munich; PAR: Paris). Source data are provided as a Source Data file.

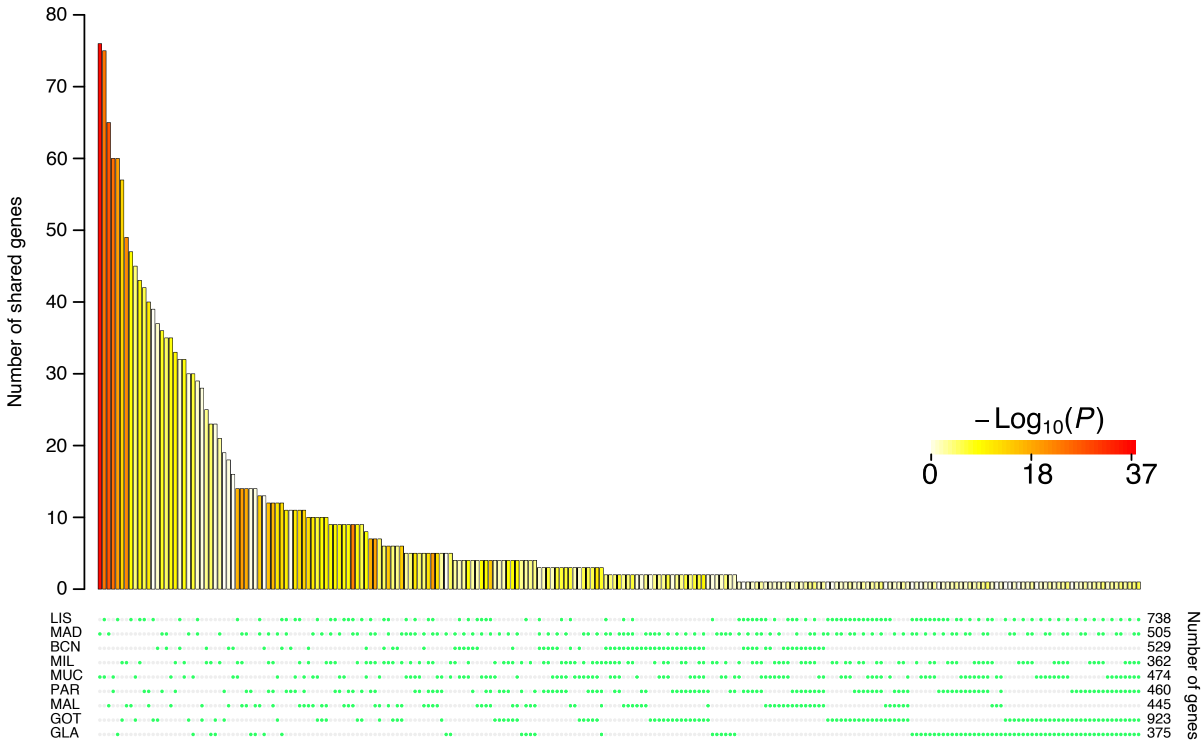

Supplementary Fig. 16. Intersection of genes associated with *Rsb* selective sweep signatures. The bar plot in the top panel shows how many genomic windows are shared for each multi-way comparison, with the populations that are compared highlighted by green dots in the lower panel. The colour of the bar plot indicates the significance of the comparison, i.e., are more windows shared in the respective multi-way comparison than expected by chance (Legend shows respective p-values). The number of windows in the lower panel corresponds to the total number of outlier windows detected in each population. Bonferroni-adjusted p-values were estimated using one-sided Fisher's Exact Tests in the SuperExactTest R-package (see Methods). The population code is indicated on the left in the lower panel (BCN: Barcelona; GLA: Glasgow; GOT: Gothenburg; LIS: Lisbon; MAD: Madrid; MAL: Malmö; MIL: Milan; MUC: Munich; PAR: Paris). Source data are provided as a Source Data file.

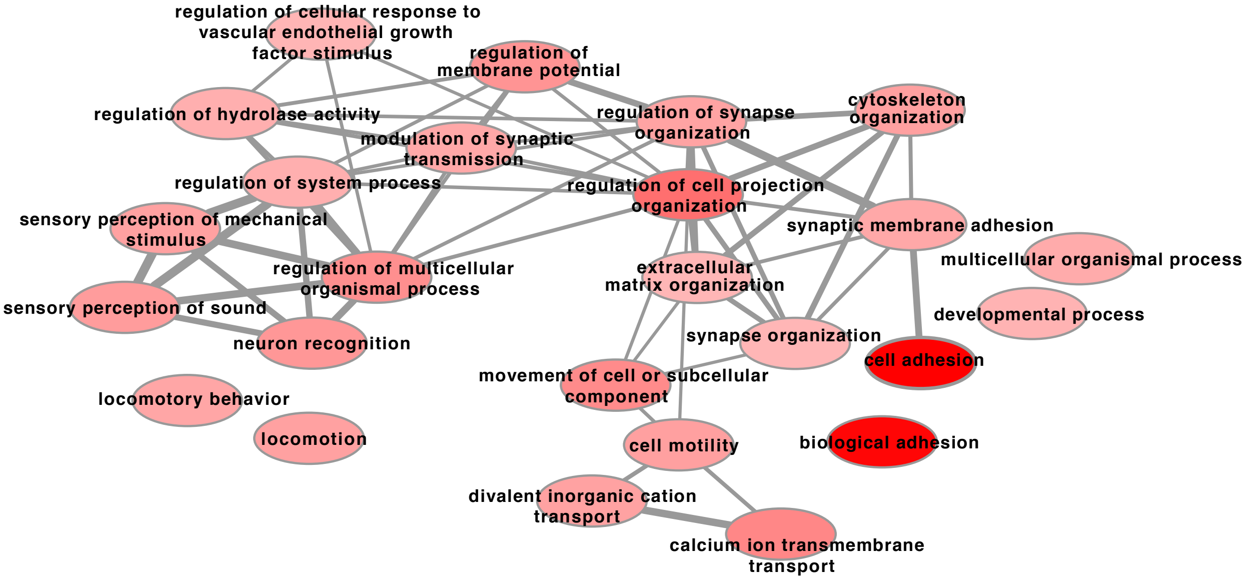

Supplementary Fig. 17. Network of urbanisation associated (LFMM) gene ontology (GO) terms (biological processes). The network shows the relationship of GO terms associated with urban-associated genes based on the number of shared genes between GO terms. The number of shared genes is given by the thickness of the grey lines. The colour intensity shows the degree of enrichment based on the p-value, with the highest intensity showing the lowest p-value. 28 GO terms were enriched at a false discovery rate (FDR) < 0.05 and 36 GO terms had an FDR > 0.05 after correction for multiple testing with FDR values ranging from 1.87×10^{-7} to 2.23×10^{-1}. One-sided p-values were estimated using hypergeometric tests in GOrilla (see Methods).

Supplementary Tables

Supplementary Table 1. Locality or urban pair (city name), year of sampling, season, centred geographical coordinates per site (urban/rural populations), urbanisation degree (PC_{Urb}, positive values indicate higher urbanisation intensity), number of genotyped individuals (n), expected heterozygosity (H_e), pairwise genetic differentiation (F_{ST}) and distance between the urban and rural populations for each of the studied localities. Cities are sorted in alphabetical order.

			Urban				Rural					
**City**	**Year**	**Season**	**Coordinates**	**PC_{Urb}**	**n**	**H_e**	**Coordinates**	**PC_{Urb}**	**n**	**H_e**	**F_{ST}**	**Distance (km)**
Barcelona	2015	Winter	41°23'24.0"N 2°11'24.0"E	2.00	10	0.341	41°42'00.0"N 2°21'36.0"E	-2.29	10	0.348	0.030	5
Glasgow	2015	Breeding	55°52'48.0"N 4°15'36.0"W	2.96	10	0.337	56°07'12.0"N 4°35'24.0"W	-1.71	10	0.344	0.030	33
Gothenburg	2015	Breeding	57°41'24.0"N 11°56'24.0"E	2.13	10	0.342	57°30'00.0"N 12°00'36.0"E	-2.39	10	0.350	0.033	25
Lisbon	2014	Post-Breeding	38°44'24.0"N 9°10'48.0"W	0.65	10	0.321	38°51'36.0"N 8°49'48.0"W	-1.86	10	0.333	0.057	33
Madrid	2014	Breeding	40°26'24.0"N 3°43'48.0"W	1.60	10	0.344	40°34'12.0"N 4°09'36.0"W	-2.05	10	0.348	0.031	39
Malmö	2013	Breeding	55°36'00.0"N 12°59'24.0"E	2.32	16	0.356	55°39'00.0"N 13°34'12.0"E	-2.44	16	0.355	0.020	37
Milan	2014	Post-Breeding	45°31'48.0"N 9°12'36.0"E	1.49	10	0.342	45°49'12.0"N 9°17'24.0"E	-2.24	10	0.343	0.040	34
Munich	2015	Winter	48°07'48.0"N 1°34'12.0"E	3.39	10	0.350	47°58'12.0"N 11°14'24.0"E	-1.36	10	0.351	0.025	30
Paris	2014	Breeding	48°52'12.0"N 2°10'48.0"E	2.36	10	0.351	48°18'00.0"N 2°39'36.0"E	-2.58	10	0.351	0.025	70

Notes: Expected heterozygosity is significantly lower in urban compared to rural great tits in Glasgow, Gothenburg Barcelona, Madrid and Lisbon based on t-tests ($P < 0.05$).

Supplementary Table 2. Pairwise genetic differentiation (F_{ST}, lower triangle) with permutation-based p-values (upper triangle). Estimates based on a randomly thinned SNP dataset with 21,062 SNPs. BCN: Barcelona; GLA: Glasgow; GOT: Gothenburg; LIS: Lisbon; MAD: Madrid; MAL: Malmö; MIL: Milan; MUC: Munich; PAR: Paris. UR: Urban population; RU: Rural populations.

	GLA_UR	***MIL_RU***	***MIL_UR***	***GOT_UR***	***MAL_RU***	***PAR_UR***	***MAD_UR***	***MUC_RU***	***MAL_UR***	***LIS_RU***	***BCN_RU***	***MAD_RU***	***MUC_UR***	***GLA_RU***	***PAR_RU***	***BCN_UR***	***LIS_UR***	***GOT_RU***
GLA_UR	--	0.001	0.001	0.001	0.001	0.001	0.001	0.001	0.001	0.001	0.001	0.001	0.001	0.001	0.001	0.001	0.001	0.001
MIL_RU	0.037	--	0.001	0.001	0.001	0.001	0.001	0.001	0.001	0.001	0.001	0.001	0.001	0.001	0.001	0.001	0.001	0.001
MIL_UR	0.038	0.025	--	0.001	0.001	0.001	0.001	0.001	0.001	0.001	0.001	0.001	0.001	0.001	0.001	0.001	0.001	0.001
GOT_UR	0.037	0.026	0.027	--	0.001	0.001	0.001	0.001	0.001	0.001	0.001	0.001	0.001	0.001	0.001	0.001	0.001	0.001
MAL_RU	0.03	0.018	0.018	0.018	--	0.004	0.001	0.008	0.001	0.001	0.001	0.001	0.001	0.001	0.001	0.001	0.001	0.001
PAR_UR	0.025	0.013	0.013	0.013	0.005	--	0.001	0.423	0.002	0.001	0.001	0.001	0.026	0.001	0.035	0.001	0.001	0.003
MAD_UR	0.035	0.023	0.024	0.023	0.015	0.01	--	0.001	0.001	0.001	0.001	0.001	0.001	0.001	0.001	0.001	0.001	0.001
MUC_RU	0.025	0.013	0.014	0.013	0.005	0	0.01	--	0.006	0.001	0.001	0.001	0.006	0.001	0.025	0.001	0.001	0.003
MAL_UR	0.028	0.016	0.016	0.016	0.007	0.004	0.013	0.003	--	0.001	0.001	0.001	0.003	0.001	0.003	0.001	0.001	0.003
LIS_RU	0.049	0.039	0.04	0.039	0.031	0.027	0.031	0.026	0.029	--	0.001	0.001	0.001	0.001	0.001	0.001	0.001	0.001
BCN_RU	0.031	0.018	0.018	0.018	0.01	0.005	0.015	0.005	0.008	0.031	--	0.001	0.001	0.001	0.001	0.034	0.001	0.001
MAD_RU	0.029	0.018	0.018	0.018	0.01	0.005	0.01	0.004	0.008	0.025	0.01	--	0.001	0.001	0.001	0.001	0.001	0.001
MUC_UR	0.026	0.015	0.015	0.014	0.007	0.001	0.012	0.001	0.004	0.027	0.007	0.006	--	0.001	0.001	0.001	0.001	0.001
GLA_RU	0.01	0.027	0.029	0.028	0.019	0.015	0.024	0.015	0.019	0.038	0.02	0.019	0.016	--	0.001	0.001	0.001	0.001
PAR_RU	0.026	0.014	0.015	0.013	0.007	0.001	0.011	0.001	0.004	0.028	0.007	0.006	0.002	0.016	--	0.001	0.001	0.001
BCN_UR	0.04	0.027	0.029	0.029	0.02	0.016	0.025	0.015	0.019	0.04	0.01	0.02	0.016	0.029	0.016	--	0.001	0.001
LIS_UR	0.066	0.058	0.058	0.057	0.048	0.045	0.05	0.044	0.047	0.048	0.05	0.046	0.046	0.056	0.046	0.06	--	0.001
GOT_RU	0.026	0.016	0.016	0.014	0.007	0.002	0.011	0.002	0.005	0.028	0.007	0.007	0.004	0.016	0.003	0.017	0.046	--

Supplementary Table 3. Genes associated with "Core urbanisation SNPs".

Gene symbol	Gene name/ Description	Selection (N. localities)
FARP1	FERM, ARH/RhoGEF and pleckstrin domain protein 1	1
CELF2	CUGBP Elav-like family member 2	0
IQGAP2	IQ motif containing GTPase activating protein 2	1
RNF38	ring finger protein 38	2
DACH1	dachshund family transcription factor 1	0
DHCR24	24-dehydrocholesterol reductase	0
SV2C	synaptic vesicle glycoprotein 2C	1
AVL9	AVL9 cell migration associated	0
CADM2	cell adhesion molecule 2	0
GABRG3	gamma-aminobutyric acid type A receptor gamma3 subunit	0
DNAI1	dynein axonemal intermediate chain 1	2
NTRK2	neurotrophic receptor tyrosine kinase 2	4
ABCB1	ATP binding cassette subfamily B member 1	0
ELP6	elongator acetyltransferase complex subunit 6	0
ADAMTS12	ADAM metallopeptidase with thrombospondin type 1 motif 12	3

Notes: Selection ($N_{localities}$) – Number of urban populations (localities) a particular gene is under selection in based on the haplotype-based selection analysis (Rsb value)

Supplementary Table 4. Genes putatively under selection in at least five cities based on *XP-nSL* or *Rsb*. The number gives the number of cities in which a gene was detected to be under selection based on the given summary statistic. Furthermore, we also highlight in how many cities a gene overlapped with a ZF_{ST} outlier window ($ZF_{ST} > 4$) and if a gene was also associated with urbanisation-associated SNPs based on LFMM.

Gene Symbol	Gene name/Description	*XP-nSL*	*Rsb*	ZF_{ST}	LFMM
HTR7	Serotonin (5-hydroxytryptamine) receptor 7	6	0	0	n
CDH18	Cadherin 18	6	1	5	y
LOC107206451	NA	6	0	0	n
LOC107206452	NA	6	0	0	n
PRMT3	Protein arginine N-methyltransferase 3	6	0	0	n
DLG2	Disks large homolog 2	5	0	0	y
PLEKHM3	Pleckstrin homology domain-containing family M member 3	5	1	0	n
CARF	Calcium-responsive transcription factor	5	0	0	n
ANKRD27	Ankyrin repeat domain-containing protein 27	5	1	1	n
DPY19L3	Probable C-mannosyltransferase DPY19L3	5	0	0	n
LOC107209655	neural-cadherin-like	5	0	0	n
PDCD5	Programmed cell death protein 5	5	0	0	n
CH25H	Cholesterol 25-hydroxylase	5	0	0	n
LOC107206916	interferon-induced protein with tetratricopeptide repeats 5-like	5	0	0	n
LOC107206998	putative lysosomal acid lipase/cholesteryl ester hydrolase	5	0	0	n
LOC107207000	NA	5	0	0	n
SLC16A12	Monocarboxylate transporter 12	5	0	0	n
STK38L	Serine/threonine-protein kinase 38-like	5	1	0	n
NRXN3	Neurexin-3-beta	5	0	0	y
TDP1	Tyrosyl-DNA phosphodiesterase 1	5	1	0	n
ANKRD1	Ankyrin repeat domain-containing protein 1	5	0	0	n
LOC107206848	ncRNA	5	0	0	n

RPP30	Ribonuclease P protein subunit p30	5	0	0	n
DBX1	Homeobox protein DBX1	5	0	0	n
NAV2	Neuron navigator 2	5	1	0	n
RBFOX3	RNA binding protein fox-1 homolog 3	5	0	0	n
KIF20B	Kinesin-like protein KIF20B	5	0	0	n
PANK1	Pantothenate kinase 1	5	0	0	n
SPATS2L	SPATS2-like protein	5	1	0	n
SORCS2	VPS10 domain-containing receptor SorCS2	5	0	0	y
TRIO	TRIO and F-actin-binding protein	5	0	0	n
DNAH5	Dynein heavy chain 5, axonemal	5	0	0	y
PTPRD	Protein Tyrosine Phosphatase, Receptor Type D	5	2	0	y
DGKZ	Diacylglycerol kinase zeta	5	2	0	n
C7H19orf73	chromosome 7 C19orf73 homolog	5	0	2	n
METTL8	mRNA N(3)-methylcytidine methyltransferase METTL8	5	0	2	n
TLK1	Serine/threonine-protein kinase tousled-like 1	5	0	2	n
PARK2	E3 ubiquitin-protein ligase parkin	5	2	0	y
APBB2	Amyloid-beta A4 precursor protein-binding family B member 2	5	0	0	n
CHRM3	Muscarinic acetylcholine receptor M3	5	0	0	n
UCHL1	Ubiquitin carboxyl-terminal hydrolase isozyme L1	5	0	0	n
SASH1	SAM and SH3 domain-containing protein 1	5	0	0	y
*GMDS **	GDP-mannose 4,6 dehydratase	1	7	0	n
*SLC6A15 **	Sodium-dependent neutral amino acid transporter B(0)AT2	0	6	0	n
LOC107206391	ncRNA	2	6	0	n
VRK1	Serine/threonine-protein kinase VRK1	2	6	0	n
LRRIQ1	Leucine-rich repeat and IQ domain-containing protein 1	0	5	0	y
C4H4orf33	chromosome 4 C4orf33 homolog	0	5	0	y

JADE1	Protein Jade-1	0	5	0	n
LOC107202860	NA	0	5	0	n
SCLT1	Sodium channel and clathrin linker 1	0	5	0	n
LOC107202827	ncRNA	0	5	0	n
MGARP	Protein MGARP	0	5	0	n
NAA15	N-alpha-acetyltransferase 15, NatA auxiliary subunit	0	5	0	y
NDUFC1	NADH dehydrogenase [ubiquinone] 1 subunit C1, mitochondrial	0	5	0	n
PAPOLA	Poly(A) polymerase alpha	3	5	0	n
GRM7	Metabotropic glutamate receptor 7	1	5	0	y

Note: Genes marked with an asterisk were differentially expressed between urban and rural great tits from Malmö in Watson *et al*. 2017.

Supplementary Table 5. Overrepresented GO terms for genes associated with urbanisation in the LFMM and BayPass analysis.

GO category	GO.ID	Description	Obs.	Fold enriched	p-value	FDR
**Processes**	GO:0098742	cell-cell adhesion via plasma-membrane adhesion molecules	38	2.920	8.27E-10	7.03E-07
**Processes**	GO:0050808	synapse organization	55	2.078	1.04E-07	4.43E-05
**Processes**	GO:0050803	regulation of synapse structure or activity	35	2.312	1.86E-06	4.36E-04
**Processes**	GO:0016358	dendrite development	36	2.272	2.05E-06	4.36E-04
**Processes**	GO:0042391	regulation of membrane potential	52	1.907	3.50E-06	5.95E-04
**Processes**	GO:0050954	sensory perception of mechanical stimulus	24	2.399	4.06E-05	5.03E-03
**Processes**	GO:0019932	second messenger-mediated signalling	46	1.823	4.14E-05	5.03E-03
**Processes**	GO:0031346	positive regulation of cell projection organization	46	1.811	4.94E-05	5.25E-03
**Processes**	GO:0010975	regulation of neuron projection development	56	1.691	5.63E-05	5.32E-03
**Processes**	GO:0022604	regulation of cell morphogenesis	52	1.650	1.96E-04	1.63E-02
**Functions**	GO:0045503	dynein light chain binding	8	4.440	1.98E-04	3.08E-02
**Functions**	GO:0030507	spectrin binding	8	4.230	2.95E-04	3.08E-02
**Functions**	GO:0050839	cell adhesion molecule binding	52	1.610	3.34E-04	3.08E-02
**Functions**	GO:0005201	extracellular matrix structural constituent	23	2.090	4.86E-04	3.36E-02
**Functions**	GO:0019199	transmembrane receptor protein kinase activity	15	2.520	6.22E-04	3.44E-02
**Functions**	GO:0019838	growth factor binding	19	2.200	7.95E-04	3.67E-02
**Functions**	GO:0005518	collagen binding	12	2.560	1.85E-03	7.33E-02
**Functions**	GO:0051959	dynein light intermediate chain binding	7	3.530	2.40E-03	8.07E-02
**Functions**	GO:0008066	glutamate receptor activity	7	3.380	3.18E-03	8.07E-02
**Functions**	GO:0045505	dynein intermediate chain binding	7	3.380	3.18E-03	8.07E-02
**Component**	GO:0097060	synaptic membrane	67	2.272	5.21E-11	8.86E-09
**Component**	GO:0098984	neuron to neuron synapse	52	2.208	2.45E-08	2.08E-06
**Component**	GO:0099572	postsynaptic specialization	48	2.061	7.54E-07	4.27E-05
**Component**	GO:0098978	glutamatergic synapse	48	1.924	5.68E-06	2.00E-04
**Component**	GO:0044309	neuron spine	28	2.450	5.89E-06	2.00E-04
**Component**	GO:0033267	axon part	47	1.839	2.43E-05	6.89E-04
**Component**	GO:0042383	sarcolemma	22	2.473	5.06E-05	1.23E-03
**Component**	GO:0098793	presynapse	53	1.692	7.69E-05	1.64E-03
**Component**	GO:0031594	neuromuscular junction	15	2.915	1.14E-04	2.16E-03
**Component**	GO:0043235	receptor complex	43	1.712	2.87E-04	4.88E-03

Notes: Obs. (Total) – observed number of genes associated with gene ontology term.
