# Supplementary material for: Continent-wide genomic signatures of adaptation to urbanisation in a songbird across Europe
Source: Nat Commun. 2021 May 20;12:2983. doi: 10.1038/s41467-021-23027-w (PMC8137928; doi:10.1038/s41467-021-23027-w)
Supplement: Supplementary file 3 — Reporting Summary [file 41467_2021_23027_MOESM3_ESM.pdf]

nature research

Corresponding author(s): Dr. Pablo Salmón; Dr. Caroline Isaksson

Last updated by author(s): Mar 31, 2021

Reporting Summary

Nature Research wishes to improve the reproducibility of the work that we publish. This form provides structure for consistency and transparency in reporting. For further information on Nature Research policies, see our Editorial Policies and the Editorial Policy Checklist.

Statistics

For all statistical analyses, confirm that the following items are present in the figure legend, table legend, main text, or Methods section.

n/a	Confirmed	
☐	☒	The exact sample size (*n*) for each experimental group/condition, given as a discrete number and unit of measurement
☐	☒	A statement on whether measurements were taken from distinct samples or whether the same sample was measured repeatedly
☐	☒	The statistical test(s) used AND whether they are one- or two-sided<br>*Only common tests should be described solely by name; describe more complex techniques in the Methods section.*
☒	☐	A description of all covariates tested
☐	☒	A description of any assumptions or corrections, such as tests of normality and adjustment for multiple comparisons
☐	☒	A full description of the statistical parameters including central tendency (e.g. means) or other basic estimates (e.g. regression coefficient) AND variation (e.g. standard deviation) or associated estimates of uncertainty (e.g. confidence intervals)
☐	☒	For null hypothesis testing, the test statistic (e.g. *F*, *t*, *r*) with confidence intervals, effect sizes, degrees of freedom and *P* value noted<br>*Give P values as exact values whenever suitable.*
☒	☐	For Bayesian analysis, information on the choice of priors and Markov chain Monte Carlo settings
☒	☐	For hierarchical and complex designs, identification of the appropriate level for tests and full reporting of outcomes
☐	☒	Estimates of effect sizes (e.g. Cohen's *d*, Pearson's *r*), indicating how they were calculated

Our web collection on statistics for biologists contains articles on many of the points above.

Software and code

Policy information about availability of computer code

Data collection	UrbanizationScore image-analysis software; Axiom Analysis Suite v.1.1.0.616 (Affymetrix); Plink v.1.9 .
Data analysis	VCFtools; Plink v.1.9 ; fastStructure v.1.0 ; Treemix v.1.3; EEMS; LFMM v.1.5; BayPass v.2.1; Genodive v.3; fastPHASE v.1.4; selscan v.1.3.0.; haplostrips software v.1.3; BEDtools v.2.28; WebGestalt; Cytoscape v.3.6.1; GOrilla; R v.3.6.1 (packages: UMAP v.0.2.7.0, WindowScanR v.0.1, csaw v.3.12, BaylorEdPsych v.0.5, SNPrelate v.3.12, plyranges v.3.12, GenomicRanges v.3.12).

For manuscripts utilizing custom algorithms or software that are central to the research but not yet described in published literature, software must be made available to editors and reviewers. We strongly encourage code deposition in a community repository (e.g. GitHub). See the Nature Research guidelines for submitting code & software for further information.

Data

Policy information about availability of data

All manuscripts must include a data availability statement. This statement should provide the following information, where applicable:

- Accession codes, unique identifiers, or web links for publicly available datasets
- A list of figures that have associated raw data
- A description of any restrictions on data availability

The genotyping data that support the findings of this study is available in variant call format (VCF) via the European Variation Archive (EVA) with the accession number PRJEB44069. The source data for Figs. 1b, 2a, 2c, 2d, 3b, 3c, 3d, 4b, 4c, 4e and Supplementary Figs. 1, 10, 12, 14, 15 and 16 are provided as a Source Data file.

Field-specific reporting

Please select the one below that is the best fit for your research. If you are not sure, read the appropriate sections before making your selection.

☐ Life sciences ☐ Behavioural & social sciences ☒ Ecological, evolutionary & environmental sciences

For a reference copy of the document with all sections, see nature.com/documents/nr-reporting-summary-flat.pdf

Ecological, evolutionary & environmental sciences study design

All studies must disclose on these points even when the disclosure is negative.

Study description	Investigation of the evolutionary response to urbanisation, using the great tit (Parus major) in nine urban/rural-pairs of populations across Europe. 192 individuals were genotyped and 517,603 SNPs across the whole genome was used for the genomic analyses.
Research sample	We sampled a total of 192 great tits (aged > 1 year old) with 10-16 individuals per habitat (see details regarding specific sample sizes in the Supplementary Table 1). This way we increased the spatial resolution of the study without loosing power per urban center. In addition, selecting preferably birds of 1 year or more increases the certainty that the individuals are resident, i.e., urban or rural, and have experienced each habitat-specific selective pressures.
Sampling strategy	Birds were captured in their nest during the nestling rearing period or using mist netting. We use 10 or more individuals per habitat in order to maximize the spatial resolution of the study.
Data collection	Data were collected by P.S., C.B., N.J.D., D.M.D., B.H., J.P.T., J.C.S., and Ph.S. Birds were capture using mist nets or trapping them in their nest-boxes during the breeding season. Blood samples (approx. 25 µl) were obtained either from the jugular or brachial vein and stored at 4 ºC in ethanol or SET buffer and subsequently frozen at -20 ºC.
Timing and spatial scale	All the individuals used in the study were sampled between June 2013 and December 2015 from 9 paired populations (urban/rural: Barcelona-January to March 2015-, Glasgow -March to June 2015-, Gothenburg -June 2015-, Lisbon -September 2014-, Madrid -March to September 2014-, Malmö -June 2013 and March 2014-, Milan -September 2014-, Munich -December 2015- and Paris -April to May 2014-; 18 theoretical populations in total). In all cases urban and rural individuals were captured within the same season/year, usually around the breeding season to ensure that birds were resident individuals in each habitat. Barcelona and Munich were sampled during winter period, however, in both cases only known birds (recaptures) were included in the study, thus, all birds can be considered resident.
Data exclusions	No data was excluded from the analyses.
Reproducibility	In order to have reproducibility of the results , the study is based on nine independent replicates of urban/rural population pairs. No population bias were observed regarding the genotyping of the individuals.
Randomization	During genotyping, samples were randomized by locality and habitat between two plates, in order to avoid any bias during the process. Randomization was also applied in certain analyses, see Methods section for detailed description.
Blinding	Blinding was not necessary as all statistics were directly computed from phenotypic, ecological and genomic data without knowing the directionality of the outcome.

Did the study involve field work? ☒ Yes ☐ No

Field work, collection and transport

Field conditions	Field work was carried out avoiding extreme weather conditions, i.e., strong rainfall or low temperatures, to ensure the wellbeing of the animals. No data on temperature or rainfall was recorded during the field work as this was not an objective of the study.
Location	Field work was carried out in nine localities across Europe. Detailed information regarding coordinates, urbanisation intensity and year of sampling are available in Supplementary Table 1.
Access & import/export	Authorities (e.g., municipalities) and private landowners have agreed for us to catch birds on their properties. Import permit dnr:6.7.18-3398/15.
Disturbance	During sampling, the handling time for each bird was minimized and standard protocols were applied to obtain the biological samples (maximum of 15 minutes between capture and sampling), therefore, ensuring the welfare of the individuals. During the mist netting sessions the nets were constantly visually monitored and no sessions were performed under extreme weather conditions.

Reporting for specific materials, systems and methods

We require information from authors about some types of materials, experimental systems and methods used in many studies. Here, indicate whether each material, system or method listed is relevant to your study. If you are not sure if a list item applies to your research, read the appropriate section before selecting a response.

Materials & experimental systems

n/a	Involved in the study
☒	☐ Antibodies
☒	☐ Eukaryotic cell lines
☒	☐ Palaeontology and archaeology
☐	☒ Animals and other organisms
☒	☐ Human research participants
☒	☐ Clinical data
☒	☐ Dual use research of concern

Methods

n/a	Involved in the study
☒	☐ ChIP-seq
☒	☐ Flow cytometry
☒	☐ MRI-based neuroimaging

Animals and other organisms

Policy information about studies involving animals; ARRIVE guidelines recommended for reporting animal research

Laboratory animals	The study did not involve laboratory animals.
Wild animals	Adult (1 year old or more) great tit (Parus major) were captured in their nest during the nestling rearing period, using mist netting or during night nest box monitoring. Both males and females were used in this study and the sexes were balanced across populations.
Field-collected samples	Immediately after their capture birds were kept in a cloth bag to avoid disturbances until the blood sample was taken either from the jugular or brachial vein (a maximum of 15 minutes between capture and sampling). All birds used in the study were released once recovered from the sampling protocol (total time of the protocol approximately 20-30 minutes). Blood samples (approx. 25 µl) were obtained either from the jugular or brachial vein and stored at 4 ºC in ethanol or SET buffer and subsequently frozen at -20 ºC.
Ethics oversight	Ethical permits were obtained from all countries and local authorities: Spain (Barcelona): Environment Department of the Generalitat de Catalunya permit no. AECC/SF/0438 to J.C.S.; UK (Glasgow): Scottish Natural Heritage (permit no. 52463) and UK Home Office license 70/7899 to B.H.; Sweden (Gothenburg and Malmö): the Malmö-Lund animal Ethical Committee permit no. M454 12:1 to C.I.; Portugal (Lisbon): CEMPA and Portuguese Ministry of Environment (ICNF) credential nos. 40/2014 and 164/2014 to C.I. and P.S.; Spain (Madrid): Ministry of the Environment, Housing and Territorial Planning of Madrid permits nos. 10/103329.9/14 and 10/169940.9/13 to P.S., and nos. 10/045383.9/14, 10/127641.9/14 and 10/055393.9/14 to J.P.T.; Italy (Milan): Institute for Environmental Protection and Research (ISPRA) int. ref. nos. 15510 and 15944 and permit no. 3462 of the region of Lombardy to P.S.; Germany (Munich): the Tierschutzgesetz (TierSchG, German animal protection law) and the Regierung von Oberbayern permits nos. 55.2-1-54-2532.2-7-07 and 55.2-1-54-2532-140-11 to N.D. and Ph.S.; France (Paris): the Minister of Higher Education, Research and Innovation (Ethics Committee for Animal Experimentation license no. 005 and permit no. APAFIS#19941-2019032516275025), the Prefect of Paris and the Prefect of Seine et Marne (permit nos. DRIEE-2012-31 and DRIEE-2012-32) and the CRBPO (National Museum of Natural History, permit no. 537 and ringing license no. 1454) to C.B.

Note that full information on the approval of the study protocol must also be provided in the manuscript.
